# Interdental Papilla Length and the Perception of Aesthetics in Asymmetric Situations

**DOI:** 10.1155/2015/125146

**Published:** 2015-06-03

**Authors:** Yung Cheng Paul Yu, Ahmed Alamri, Helena Francisco, Sang-Choon Cho, Stuart Hirsch

**Affiliations:** Ashman Department of Periodontology and Implant Dentistry, New York College of Dentistry, 345 East 24th Street, Suite 3W, New York, NY 10010, USA

## Abstract

The purpose of the study was to determine if there was a difference in the perception of aesthetics, by dental specialty, using computer assisted asymmetric alteration of the papilla length in the aesthetic zone with an apical alteration of the contact point of the clinical crowns. Standardized photographs were presented to sixty-five randomly selected dentists from New York University College of Dentistry on a computer screen for evaluation. Then, the dental professionals were asked to rate the smile in each picture. Control and experiment photographs were used. Data was analyzed using the statistical package SPSS version 21 and one-way ANOVA. The perception of esthetics depends on the dental professional specialty; results provide evidence that asymmetric deficiency in papilla length of 2 mm or more is perceived as “unattractive” by the dental specialists.

## 1. Introduction

Over the past 30 years, replacing missing teeth with dental implants has become a viable solution to conventional fixed or removable prosthodontics [1]. However, the rehabilitation with implant supported prosthesis remains challenging particularly in the esthetic areas. The esthetic area is defined as the visible area during functioning and includes the anterior maxillary and mandibular teeth. Implant survival in these areas may reach 82.94% [2], while implant success varies significantly [3] reaching at times only 51.97% and even lower [2, 3]. The discrepancy between implant survival and success is not unexpected as their definitions are quite different. Implant “survival” definition is broad and encompasses all implants that are still in the mouth. The criteria of success can vary. However, it is restrictive and includes only the dental implants that present, in addition to proper integration and function, other features such as esthetic characteristics: soft tissue contours with an intact interdental papilla and a gingival outline that is harmonious with the gingival silhouette of the adjacent healthy dentition [2, 3].

One of the esthetic deficiencies occurring after implant placement is the lack of papilla between implants or between teeth and implants. The lack of the interdental papilla can lead not only to cosmetic deformities, but also to phonetic difficulty and food impaction. Therefore, achieving a predictable papilla is of outmost importance and it has been the subject of numerous studies. The vertical distance from the crest of the bone to the height of the interproximal papilla between adjacent teeth and between adjacent implants was evaluated by Tarnow et al. [4, 5]. When this distance was 5 mm or less between two adjacent teeth the papilla completely filled this space almost 100% of the time. However, the average height of tissue over the crest of bone between two adjacent implants was reported to be only 3.4 mm [4, 6] ranging from 3 to 9 mm. In addition, the anatomical features of the space between two implants are significantly different. Thus if a patient has normal interdental papilla and requires two other adjacent anterior teeth replaced, the interimplant papilla oftentimes will tend to be apical in position compared to the papilla of the adjacent teeth.

Many surgical and prosthetic techniques have been attempted to restore missed interdental papilla. However, predictable regeneration of the papilla between two adjacent dental implants remains a complex challenge [5, 7, 8]. In addition to the establishment of an anatomically correct papilla, the success of the implant rehabilitation also depends on the “perceivement” of gingival and papilla contours. Studies showed that patient and clinician perceive papilla and gingival contour differently and this difference depends on gingival symmetry. Interestingly, this “perceivement” appears to differ among dental specialties. However, there is a paucity of studies comparing the perceivement of symmetry among different dental specialties.

Clinically, the presence of the black triangle is characterized by a receded papilla visible space between the papilla and the contact point of the restorations. Whether the presence of the black triangle translates into an unfavorable esthetic outcome depends on the size of the defect as well as on the “perceivement” of this defect. If the esthetic outcome is perceived as “unfavorable” by several clinicians, then attempts should be done to rectify or prevent the defect. For example, in aesthetic demanding cases, the clinician should also consider alternative treatment plans (i.e., one implant and a cantilevered pontic) for a two-tooth edentulous space in order to achieve an improved aesthetic outcome [9].

**Figure 1:**
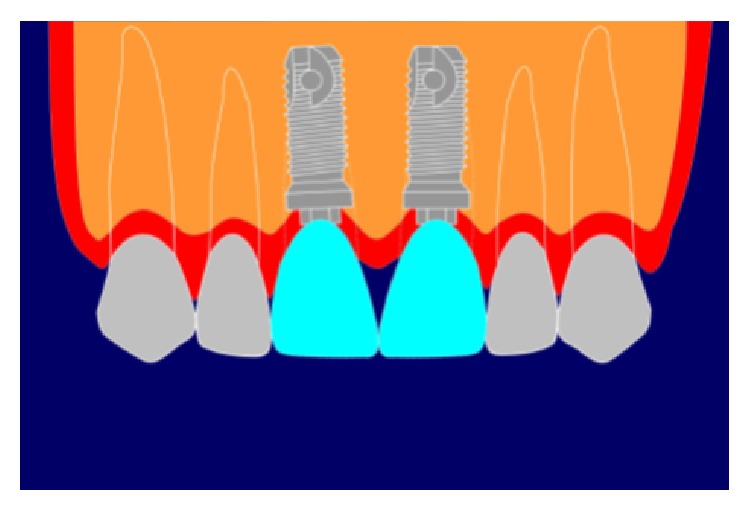
“Black triangle” between central incisors.

**Figure 2:**
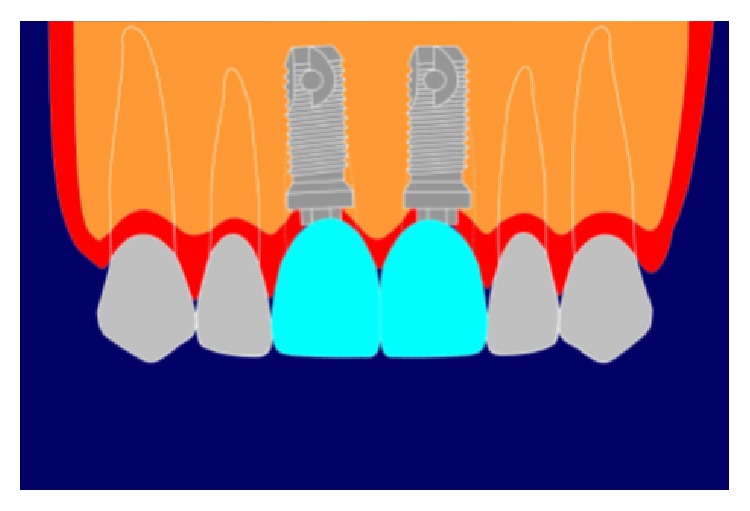
Acceptable long contact point.

**Figure 3:**
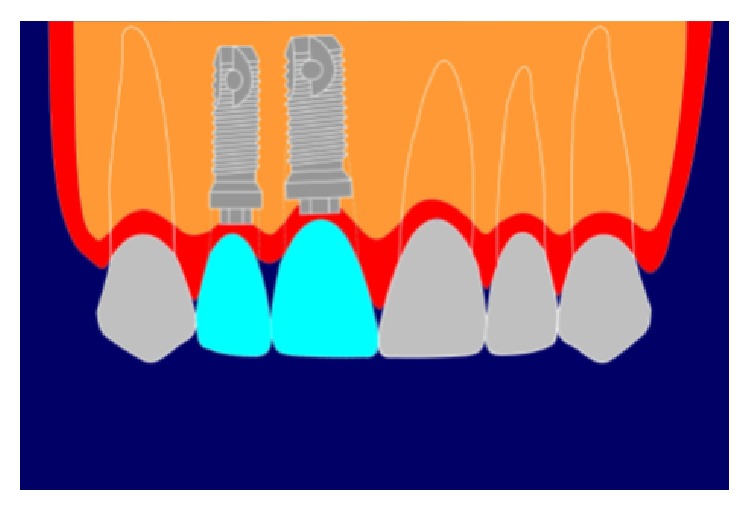
Asymmetric “black triangle.”

**Figure 4:**
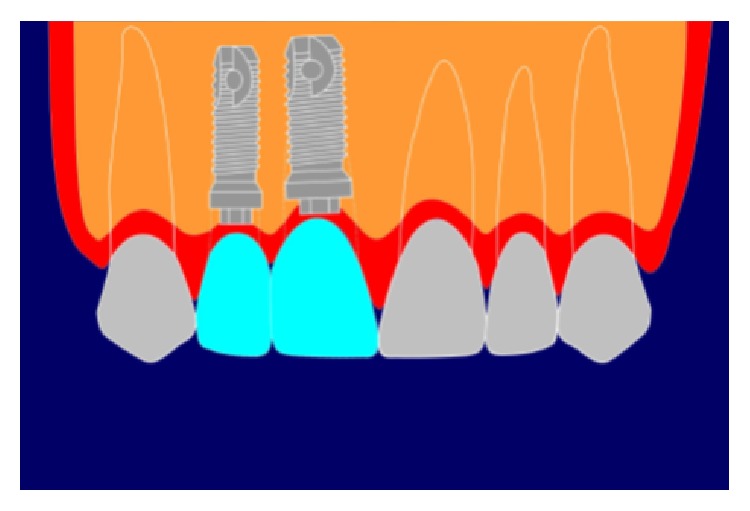
Unacceptable asymmetric long contact.

It is reported that minor alterations to teeth and surrounding tissue are discernable to dental professionals and lay people in varying degrees. Kokich Jr. et al. reported that orthodontists noted a 2 mm midline open gingival embrasure (between the central incisors) as less attractive, while lay people and general practitioners made critical note of a 3 mm open embrasure [10]. A recent study by LaVacca et al. showed that patients were not able to discern symmetric alteration of a shortened papilla length of 2 mm when soft tissue completely filled in the gingival embrasure as the contact point was relocated in an apical direction [11, 12]. To date, no studies have evaluated the influence of the asymmetric papilla length on the perception of aesthetics. Since the dental specialties emphasize different aspects of the dental care, they may also differ in their perceivement of gingival and papillary contour. We hypothesized that periodontists with their soft tissue management skills would perceive as an unfavorable outcome any deviation from normal compared to the orthodontists and general dentists. The purpose of the present study was to determine if there was a difference in the perception of aesthetics, by dental specialty. Towards this goal, we used computer assisted asymmetric alteration of the papilla length below the contact point of the clinical crowns in the aesthetic zone Figures 1, 2, 3, and 4.

## 2. Materials and Methods

### 2.1. Subjects

Sixty-five randomly selected dentists from New York University College of Dentistry participated in this study.

### 2.2. Protocol

Standardized photographs were presented to the dental professionals on a computer screen for evaluation. Then, the dental professionals were asked to rate the smile in each picture. Control and experiment photographs were used.

### 2.3. Control Photograph

**Figure 5:**
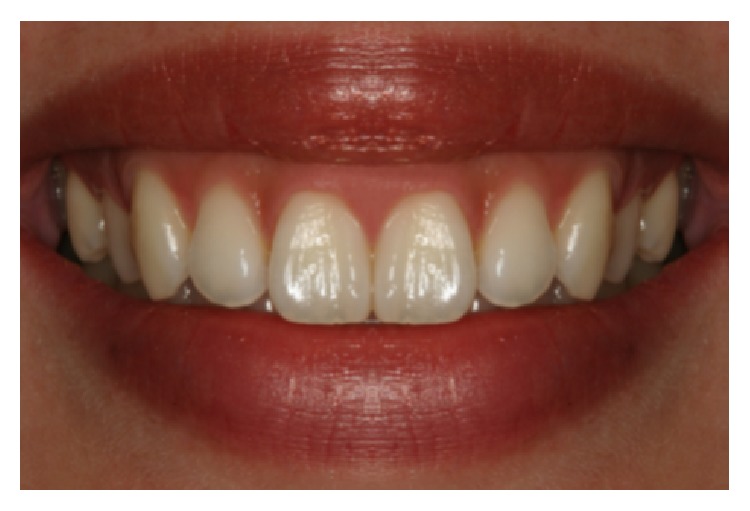
Control photograph (photo A).

A natural smile that correlated with Rufenacht's [11] tooth papilla-ideal gingival proportions was identified. A digital photograph as shown in Figure 5, limited to the lips and teeth within the smile (high smile-line), was obtained. Utilizing a computer software program (Adobe Photoshop 6.0, Adobe Systems Incorporated), the smile in the photograph was digitally enhanced. The coronal display of the papilla and gingival levels were symmetrically aligned on both sides of the arch and constituted “the gold standard” for esthetics. The purpose of this enhancement was to eliminate discrepancies and minimize any potential bias.

### 2.4. Experimental Photographs

**Figure 6:**
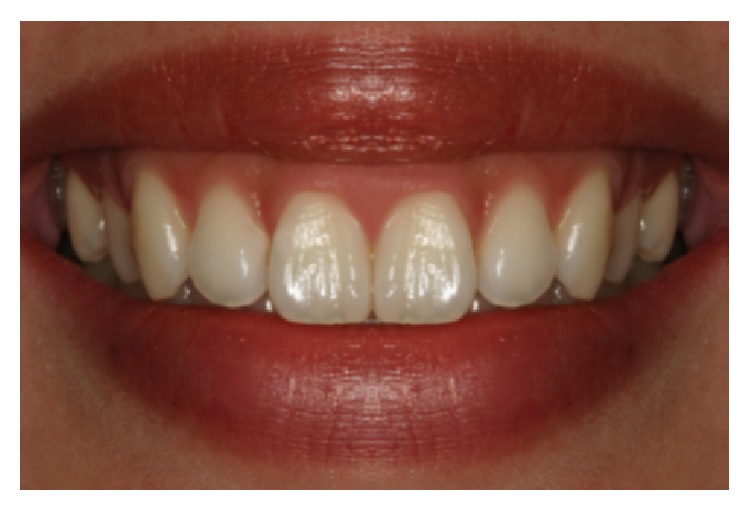
Shortened papilla b/w #7 and 8 (1 mm, photo B).

**Figure 7:**
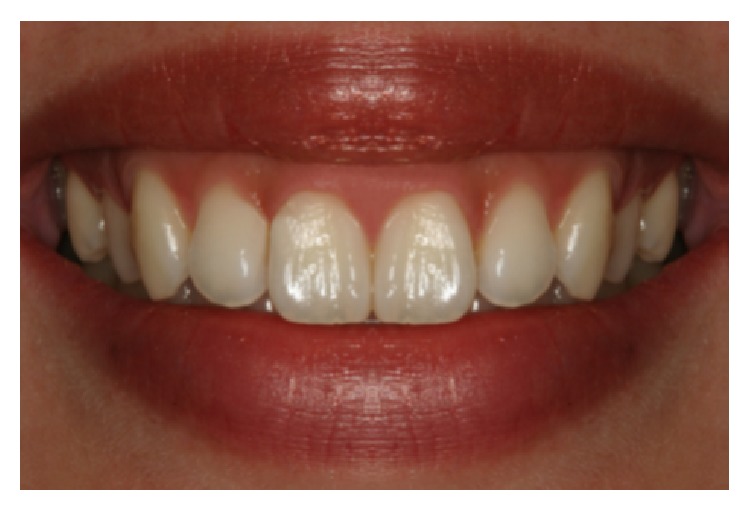
Shortened papilla b/w #7 and 8 (2 mm, photo C).

**Figure 8:**
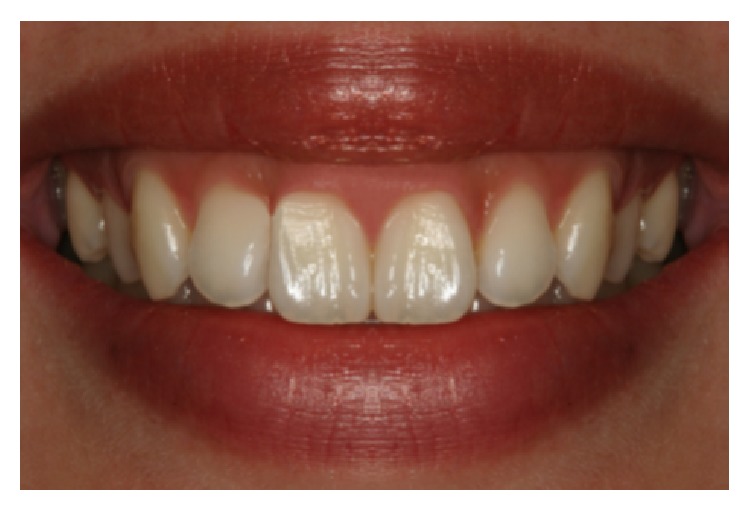
Shortened papilla b/w #7 and 8 (3 mm, photo D).

Experimental photographs were obtained by digital alterations as shown in Figures 6, 7, and 8. The location of the papilla in the control photograph was first identified and then three alterations were digitally performed. These alterations shortened asymmetrically the papilla between right central and lateral incisors incrementally by 1 mm from the position of the control. As the papilla was shortened, the crown contour and contact point between these incisors were also altered to eliminate the presence of the “black triangle” in the gingival embrasures of the photographs. Below are the photographs presented:

 photo A: control photograph;
 photo B: 1 mm shortened papilla photograph;
 photo C: 2 mm shortened papilla photograph;
 photo D: 3 mm shortened papilla photograph.

### 2.5. Perception Survey

A control and 3 altered photographs were placed on a sheet of paper. The control photograph was designated a rating order of 1. Evaluators viewed the other 3 photographs and assigned an aesthetic rating order of 1–4, according to the following scale:

1. very attractive;
2. attractive;
3. unattractive;
4. very unattractive.

### 2.6. Data Analysis

Data was analyzed using the statistical package SPSS, version 21. The ratings assigned to each photograph by the evaluators were determined and allowed for ratings comparison by specialty. Attractive and very attractive ratings were merged into a single rating “the attractive rating.” Unattractive and very unattractive ratings were also merged into “the unattractive rating.” Then, the percentages of dental professionals rating the photographs as “attractive” and “unattractive” were calculated. One-Way ANOVA was used to determine whether there were differences in the percentage of the dental professionals rating the three experimental shortened papilla photographs.

## 3. Results

### 3.1. Population Characteristics

A total of 65 dental professionals participated in this study: twenty were prosthodontists, twenty periodontists, and twenty-five general dentists.

### 3.2. The Perception of Esthetics Depends on the Dental Professional Specialty

**Figure 9:**
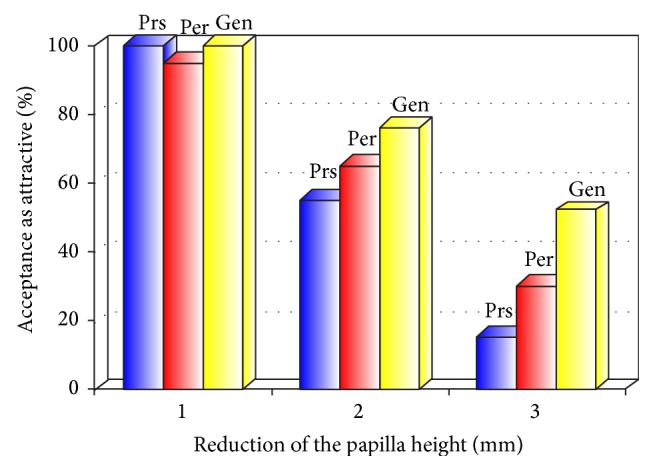
Rating of shortened papilla by specialties.

Figure 9 and Table 1 show the percentage of the dental professionals rating the smiles as attractive when the papilla was shortened by 1, 2, and 3 mm. The results show that when the papilla was shortened by 1 mm (photo B), 98% of the evaluators rated it as “attractive” with no difference among the specialists. In fact, 100% of prosthodontists, 95% of periodontists, and 100% of general dentists rated it as “attractive.” When the papilla was shortened by 2 mm (photo C), overall, 66% of the evaluators rated it as “attractive.” In fact, 55% of the prosthodontists, 65% of the periodontists, and 76% of the general dentists rated it as “attractive.” However, when the papilla was shortened by 3 mm (photo D), only 66% of evaluators rated it as “unattractive.” Among them, 85% of prosthodontists, 70% of periodontists, and 48% of general dentists rated it as “unattractive.” These results show that the perceivement of the esthetics when the papilla is shortened depends on the dental professional specialty.

**Figure 10:**
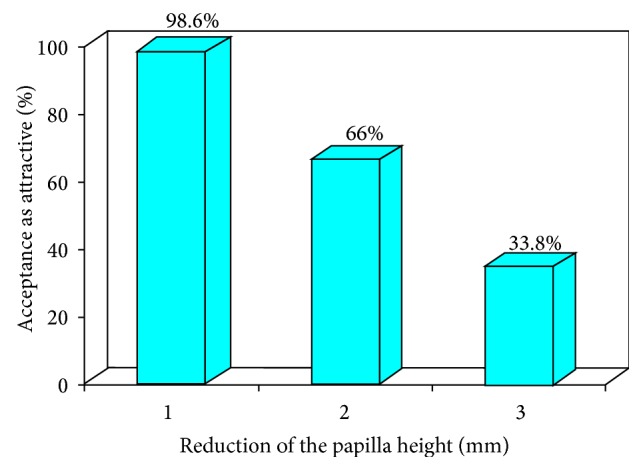
Acceptance of shortened papilla.

The esthetics is perceived as attractive only if the papilla shortening is very minor.  Figure 10 shows the ratings of “attractiveness” among all the dental professionals. Our results showed that the percentage of dental professionals rating the esthetics as “attractive” differed by the magnitude of the asymmetric papilla shortening and these results were significant (*P* = 0.002). Post hoc tests showed that these differences were significant among all the experimental papilla shortening esthetics (between 1 and 2 mm: *P* = 0.02; between 2 and 3 mm: *P* = 0.02). These results show that the esthetic perception with only 1 mm papilla shortening is rated as “attractive” by most dental professionals regardless of their specialty. However, when the papilla is shortened by 2 or 3 mm, the esthetics is rated as “attractive” by only a few dental professionals. These results provide evidence that asymmetric deficiencies in papilla length of 2 mm or more are perceived as “unattractive.”

## 4. Discussion

Within the limitations of our study that is composed of 65 dental professionals, we showed that the perceivement of esthetics compared to “the gold standard” for the interdental papilla in the esthetic zone depended on the dental professional specialty. We also found that deficiencies in the papilla as low as 2 mm were perceived as “unattractive” esthetics by most dental professionals.

In a previous study by LaVacca et al. [12], the papilla length was shortened by 2 mm bilaterally obtaining a symmetrical smile [10]. Overall, both orthodontists and patients rated this esthetic change as attractive suggesting that if no black triangles are present, patients and orthodontists perceived dental aesthetics as attractive although some variation existed. In the present study, a unilateral, asymmetrical shortening of the papilla by 2 mm was rated as unattractive by two-thirds of the total evaluators. Since some of our evaluators were orthodontists, these appears to demonstrate that, in an asymmetric situation, a 2 mm shortened papilla is more detectable compared to a symmetric situation. A 3 mm shortened papilla was considered unattractive by one-third of the evaluators. In ideal situation the lateral incisor has approximately 80% shorter clinical crown than that of the central incisor and the gingival margin is located on slightly more coronal position compared to central incisor [11]. This anatomical presentation results in a shorter papilla on the lateral incisor side than between the central incisors. Therefore, a 3 mm shortened papilla can make a lateral incisor appear squarer in form than of a central incisor. Prosthodontists appear to be more sensitive to changes in location of the contact point. As a result, they rated shortening of the papilla by 2 mm (45%) and 3 mm (85%) as unattractive when compared to periodontists (35%, 24%) and general dentists (70%, 48%), respectively. Further studies with well-characterized population will be needed to evaluate the dentist and patient perceptions regarding aesthetics and the “black triangle” and to see if changes in the papilla height between lateral and canine unilaterally and bilaterally result in similar rating by the 3 different groups of dentists.

## 5. Conclusion

Only 1.6% of evaluators rated as unattractive a papilla shortened 1 mm from the control. One-third of evaluators rated as unattractive a 2 mm shortened papilla and two-thirds of the evaluators rated as unattractive a 3 mm shortened papilla. We conclude that many dental professionals perceive even minor asymmetric shortening of the papilla unattractive. However, this is only “half” the story. Studies evaluating professionals and different populations would be needed for a more comprehensive understanding of this issue.

## Figures and Tables

**Table 1:** Rating of altered papilla by different specialties.

	0 mm				1 mm				2 mm				3 mm			
	Prs	Per	Gen	All	Prs	Per	Gen	All	Prs	Per	Gen	All	Prs	Per	Gen	All
I	20	20	25	65	2	2	9	13	0	1	3	4	0	0	1	1
II					18	17	16	51	11	12	16	39	3	6	12	21
III					0	1	0	1	9	7	6	22	14	11	8	33
IV					0	0	0	0	0	0	0	0	3	3	4	10
% of acceptance as attractive					100	95	100	98	55	65	76	66	15	30	52	33
% of acceptance as unattractive					0	5	0	1	45	35	24	33	85	70	48	66

Prs: prosthodontist, Per: periodontist, Gen: general dentist, I: very attractive, II: attractive, III: unattractive, and IV: very unattractive.
